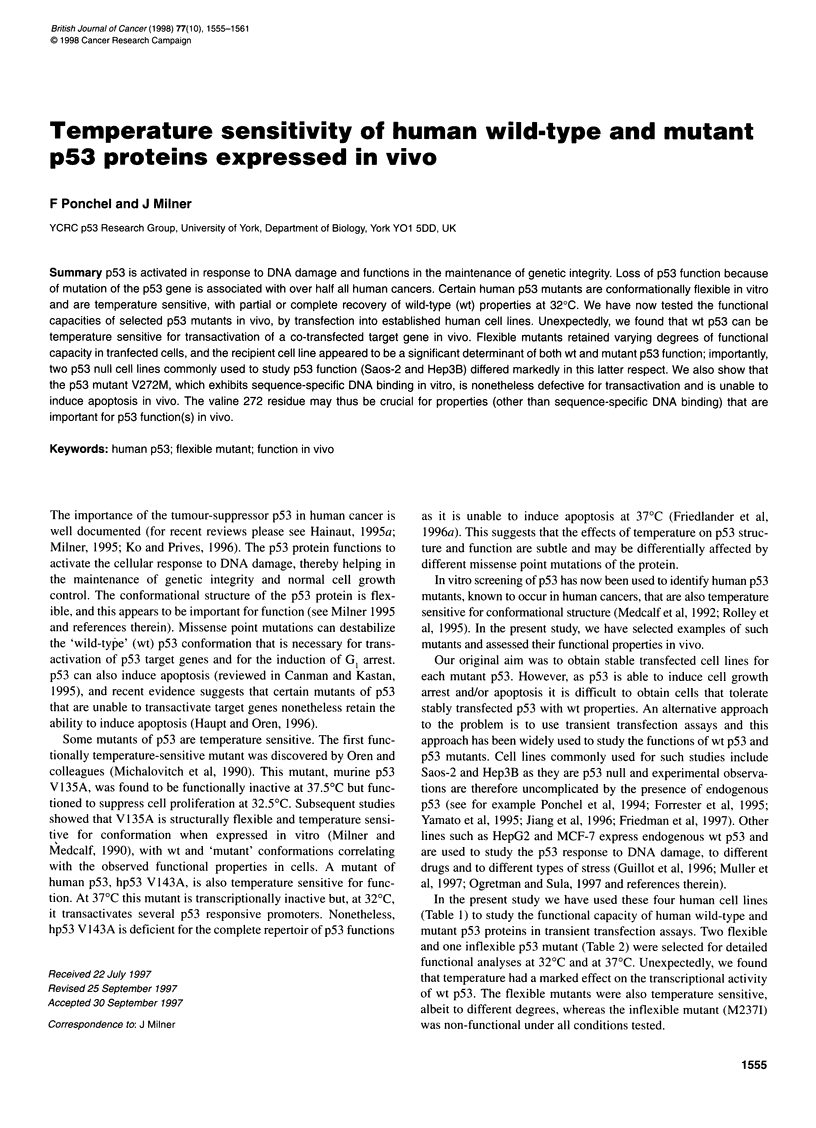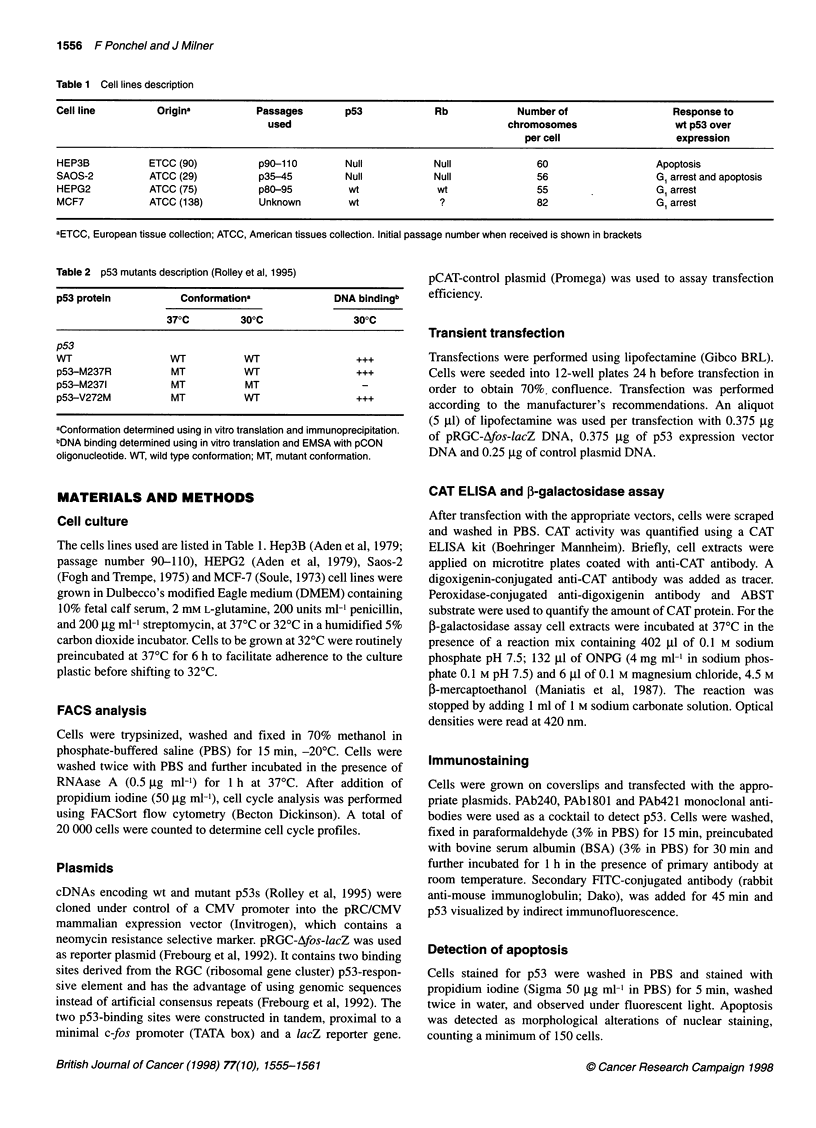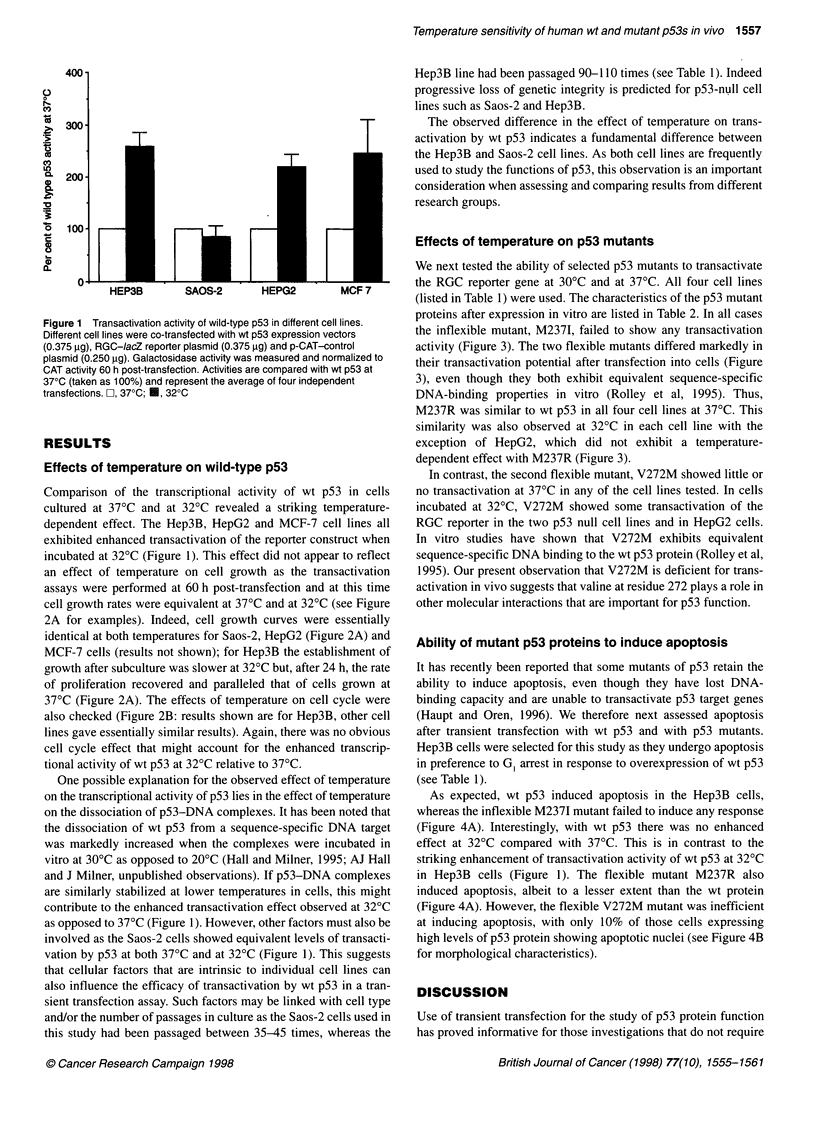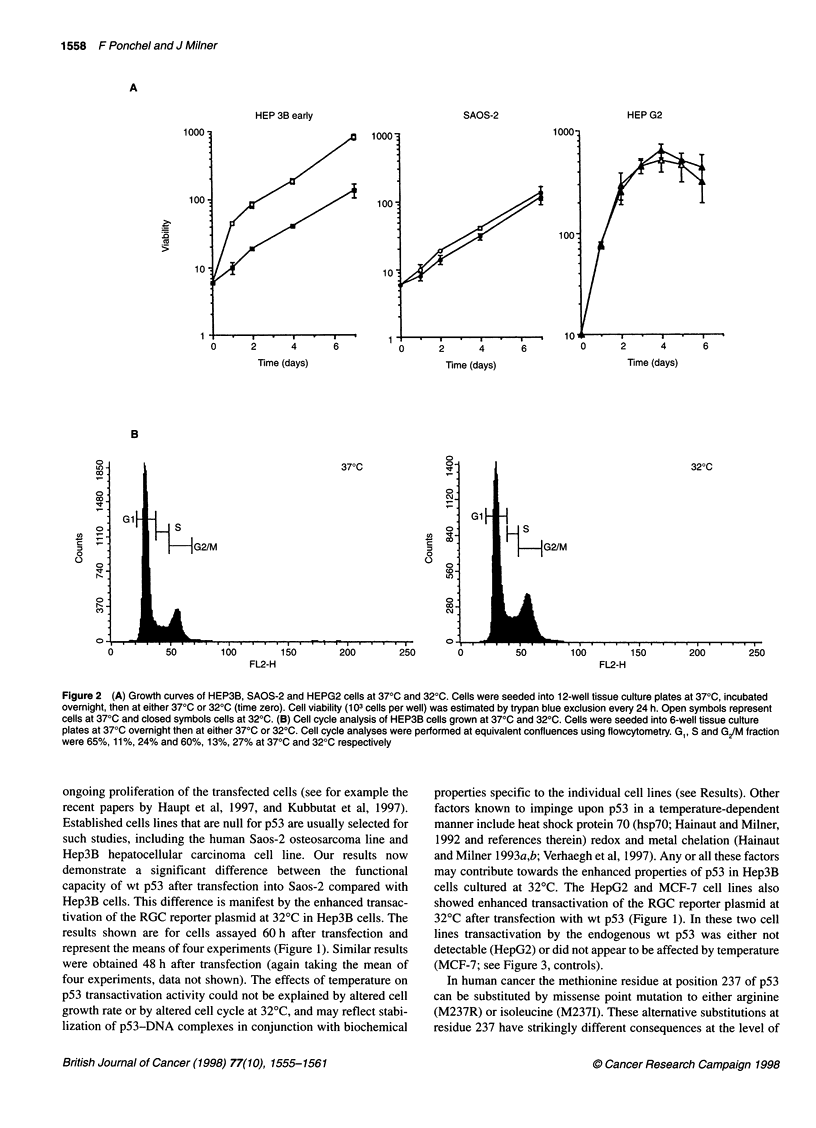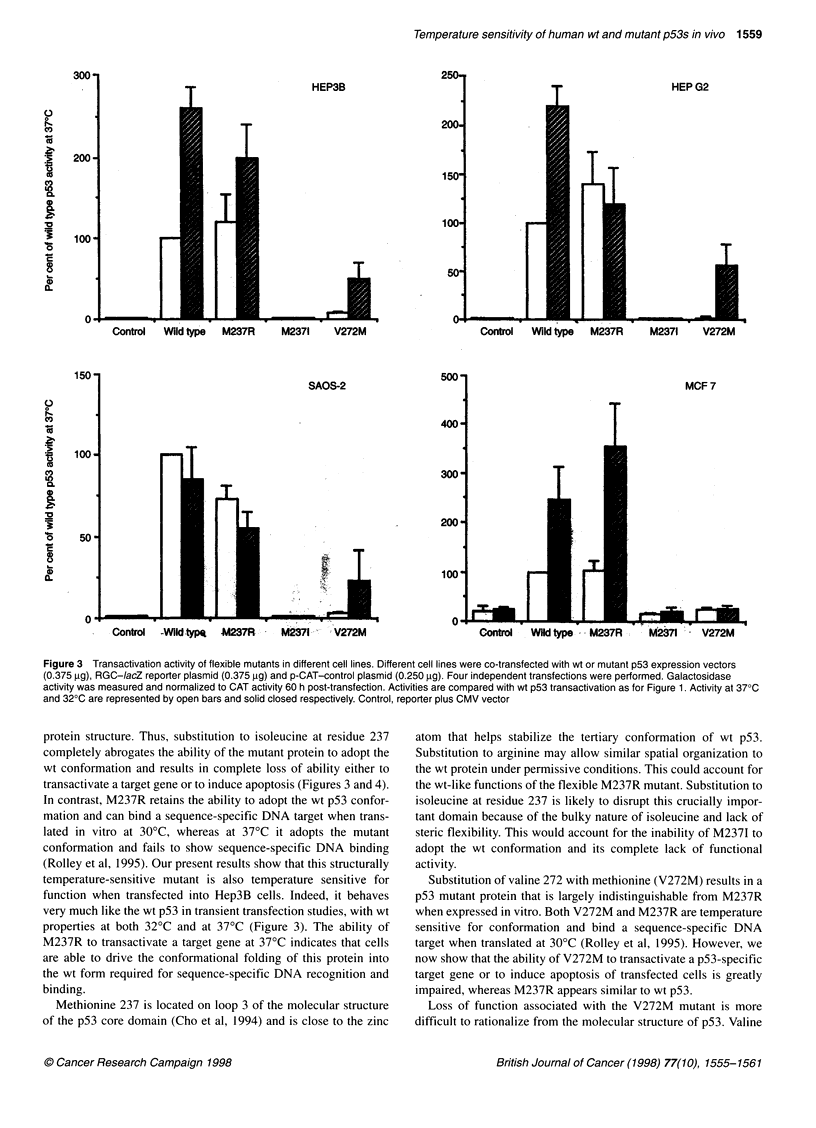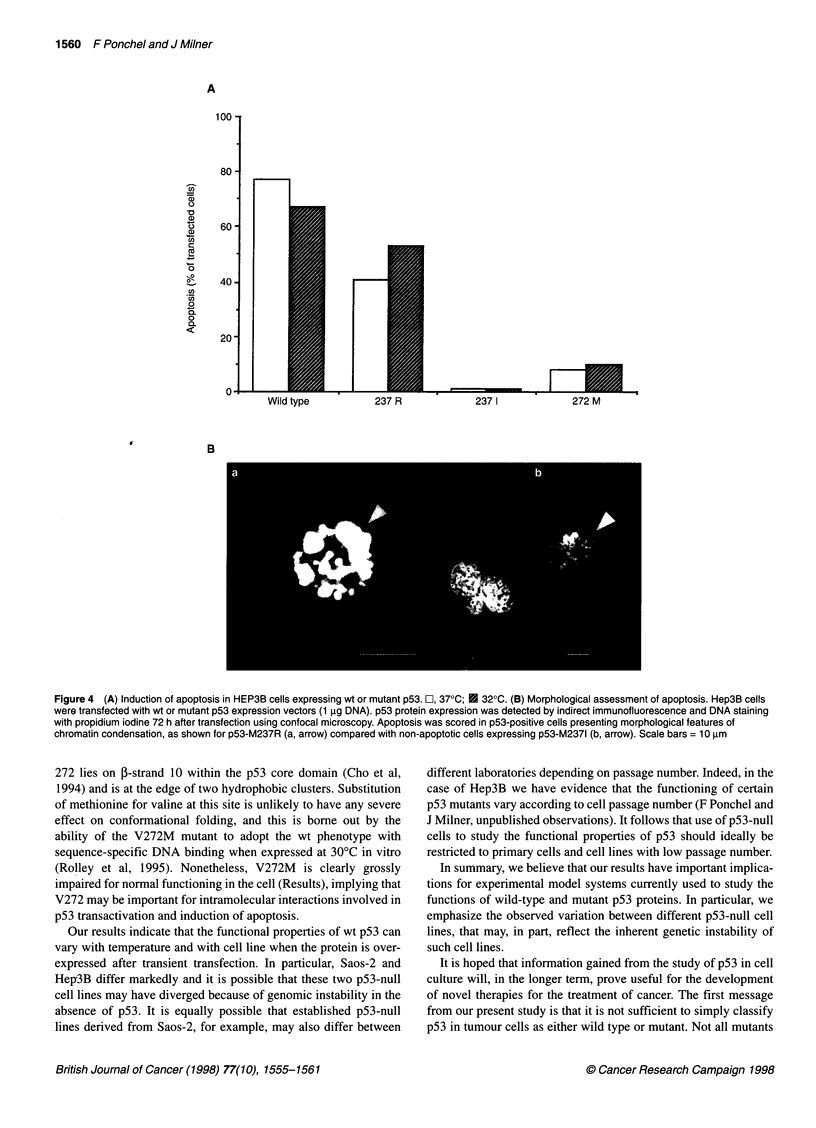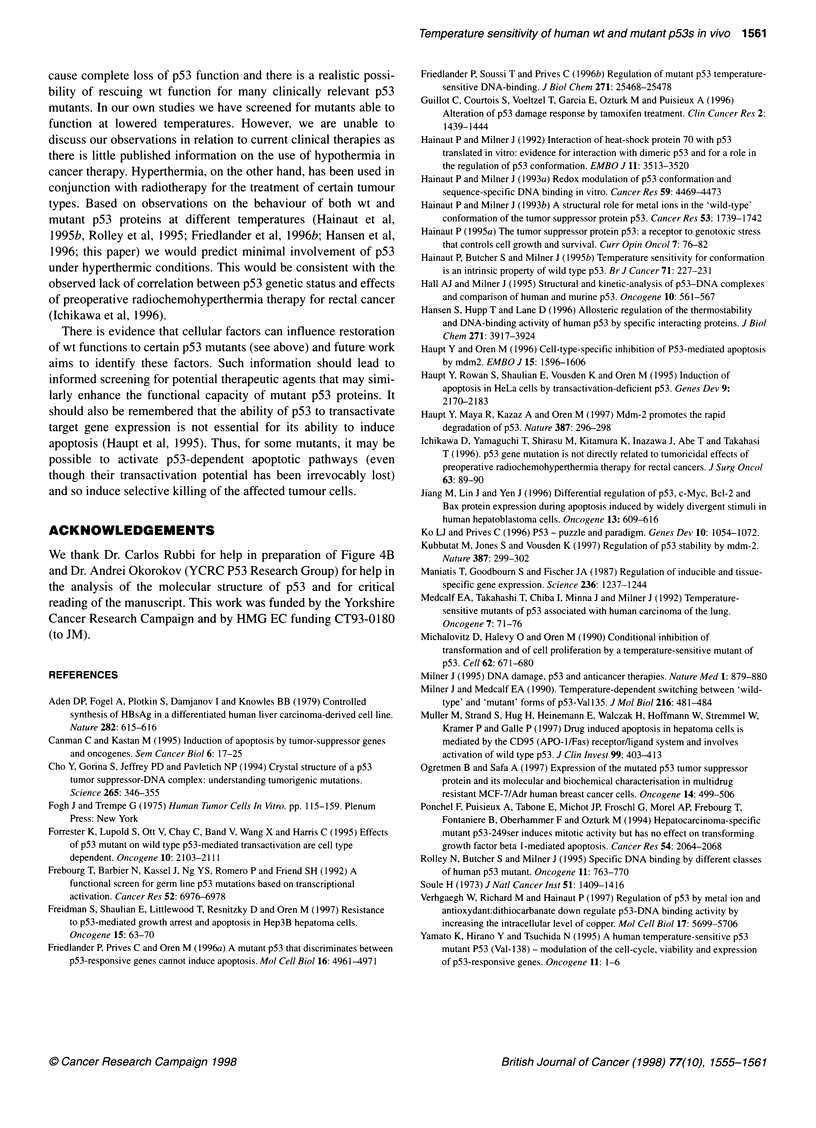# Temperature sensitivity of human wild-type and mutant p53 proteins expressed in vivo.

**DOI:** 10.1038/bjc.1998.256

**Published:** 1998-05

**Authors:** F. Ponchel, J. Milner

**Affiliations:** YCRC p53 Research Group, University of York, Department of Biology, UK.

## Abstract

**Images:**


British Journal of Cancer (1998) 77(10), 1555–1561
© 1998 Cancer Research Campaign

### Temperature sensitivity of human wild-type and mutant p53 proteins expressed in vivo

**F Ponchel and J Milner**

YCRC p53 Research Group, University of York, Department of Biology, York YO1 5DD, UK

**Summary** p53 is activated in response to DNA damage and functions in the maintenance of genetic integrity. Loss of p53 function because of mutation of the p53 gene is associated with over half all human cancers. Certain human p53 mutants are conformationally flexible in vitro and are temperature sensitive, with partial or complete recovery of wild-type (wt) properties at 32°C. We have now tested the functional capacities of selected p53 mutants in vivo, by transfection into established human cell lines. Unexpectedly, we found that wt p53 can be temperature sensitive for transactivation of a co-transfected target gene in vivo. Flexible mutants retained varying degrees of functional capacity in tranfected cells, and the recipient cell line appeared to be a significant determinant of both wt and mutant p53 function; importantly, two p53 null cell lines commonly used to study p53 function (Saos-2 and Hep3B) differed markedly in this latter respect. We also show that the p53 mutant V272M, which exhibits sequence-specific DNA binding in vitro, is nonetheless defective for transactivation and is unable to induce apoptosis in vivo. The valine 272 residue may thus be crucial for properties (other than sequence-specific DNA binding) that are important for p53 function(s) in vivo.

**Keywords:** human p53; flexible mutant; function in vivo

The importance of the tumour-suppressor p53 in human cancer is well documented (for recent reviews please see Hainaut, 1995*a*; Milner, 1995; Ko and Prives, 1996). The p53 protein functions to activate the cellular response to DNA damage, thereby helping in the maintenance of genetic integrity and normal cell growth control. The conformational structure of the p53 protein is flexible, and this appears to be important for function (see Milner 1995 and references therein). Missense point mutations can destabilize the 'wild-type' (wt) p53 conformation that is necessary for transactivation of p53 target genes and for the induction of $G_1$ arrest. p53 can also induce apoptosis (reviewed in Canman and Kastan, 1995), and recent evidence suggests that certain mutants of p53 that are unable to transactivate target genes nonetheless retain the ability to induce apoptosis (Haupt and Oren, 1996).

Some mutants of p53 are temperature sensitive. The first functionally temperature-sensitive mutant was discovered by Oren and colleagues (Michalovitch et al, 1990). This mutant, murine p53 V135A, was found to be functionally inactive at 37.5°C but functioned to suppress cell proliferation at 32.5°C. Subsequent studies showed that V135A is structurally flexible and temperature sensitive for conformation when expressed in vitro (Milner and Medcalf, 1990), with wt and 'mutant' conformations correlating with the observed functional properties in cells. A mutant of human p53, hp53 V143A, is also temperature sensitive for function. At 37°C this mutant is transcriptionally inactive but, at 32°C, it transactivates several p53 responsive promoters. Nonetheless, hp53 V143A is deficient for the complete repertoir of p53 functions as it is unable to induce apoptosis at 37°C (Friedlander et al, 1996*a*). This suggests that the effects of temperature on p53 structure and function are subtle and may be differentially affected by different missense point mutations of the protein.

In vitro screening of p53 has now been used to identify human p53 mutants, known to occur in human cancers, that are also temperature sensitive for conformational structure (Medcalf et al, 1992; Rolley et al, 1995). In the present study, we have selected examples of such mutants and assessed their functional properties in vivo.

Our original aim was to obtain stable transfected cell lines for each mutant p53. However, as p53 is able to induce cell growth arrest and/or apoptosis it is difficult to obtain cells that tolerate stably transfected p53 with wt properties. An alternative approach to the problem is to use transient transfection assays and this approach has been widely used to study the functions of wt p53 and p53 mutants. Cell lines commonly used for such studies include Saos-2 and Hep3B as they are p53 null and experimental observations are therefore uncomplicated by the presence of endogenous p53 (see for example Ponchel et al, 1994; Forrester et al, 1995; Yamato et al, 1995; Jiang et al, 1996; Friedman et al, 1997). Other lines such as HepG2 and MCF-7 express endogenous wt p53 and are used to study the p53 response to DNA damage, to different drugs and to different types of stress (Guillot et al, 1996; Muller et al, 1997; Ogretman and Sula, 1997 and references therein).

In the present study we have used these four human cell lines (Table 1) to study the functional capacity of human wild-type and mutant p53 proteins in transient transfection assays. Two flexible and one inflexible p53 mutant (Table 2) were selected for detailed functional analyses at 32°C and at 37°C. Unexpectedly, we found that temperature had a marked effect on the transcriptional activity of wt p53. The flexible mutants were also temperature sensitive, albeit to different degrees, whereas the inflexible mutant (M237I) was non-functional under all conditions tested.

*Received 22 July 1997*
*Revised 25 September 1997*
*Accepted 30 September 1997*

*Correspondence to:* J Milner

Table 1 Cell lines description

| Cell line | Origin[a] | Passages used | p53 | Rb | Number of chromosomes per cell | Response to wt p53 over expression |
|---|---|---|---|---|---|---|
| HEP3B | ETCC (90) | p90–110 | Null | Null | 60 | Apoptosis |
| SAOS-2 | ATCC (29) | p35–45 | Null | Null | 56 | $G_1$ arrest and apoptosis |
| HEPG2 | ATCC (75) | p80–95 | wt | wt | 55 | $G_1$ arrest |
| MCF7 | ATCC (138) | Unknown | wt | ? | 82 | $G_1$ arrest |

[a]ETCC, European tissue collection; ATCC, American tissues collection. Initial passage number when received is shown in brackets

Table 2 p53 mutants description (Rolley et al, 1995)

| p53 protein | Conformation[a] | | DNA binding[b] |
|---|---|---|---|
| | 37°C | 30°C | 30°C |
| *p53* | | | |
| WT | WT | WT | +++ |
| p53–M237R | MT | WT | +++ |
| p53–M237I | MT | MT | – |
| p53–V272M | MT | WT | +++ |

[a]Conformation determined using in vitro translation and immunoprecipitation. [b]DNA binding determined using in vitro translation and EMSA with pCON oligonucleotide. WT, wild type conformation; MT, mutant conformation.

### MATERIALS AND METHODS

### Cell culture

The cells lines used are listed in Table 1. Hep3B (Aden et al, 1979; passage number 90–110), HEPG2 (Aden et al, 1979), Saos-2 (Fogh and Trempe, 1975) and MCF-7 (Soule, 1973) cell lines were grown in Dulbecco's modified Eagle medium (DMEM) containing 10% fetal calf serum, 2 mM L-glutamine, 200 units ml$^{-1}$ penicillin, and 200 µg ml$^{-1}$ streptomycin, at 37°C or 32°C in a humidified 5% carbon dioxide incubator. Cells to be grown at 32°C were routinely preincubated at 37°C for 6 h to facilitate adherence to the culture plastic before shifting to 32°C.

### FACS analysis

Cells were trypsinized, washed and fixed in 70% methanol in phosphate-buffered saline (PBS) for 15 min, –20°C. Cells were washed twice with PBS and further incubated in the presence of RNAase A (0.5 µg ml$^{-1}$) for 1 h at 37°C. After addition of propidium iodine (50 µg ml$^{-1}$), cell cycle analysis was performed using FACSort flow cytometry (Becton Dickinson). A total of 20 000 cells were counted to determine cell cycle profiles.

### Plasmids cDNAs encoding wt and mutant p53s (Rolley et al, 1995) were cloned under control of a CMV promoter into the pRC/CMV mammalian expression vector (Invitrogen), which contains a neomycin resistance selective marker. pRGC-Δ*fos-lac*Z was used as reporter plasmid (Frebourg et al, 1992). It contains two binding sites derived from the RGC (ribosomal gene cluster) p53-responsive element and has the advantage of using genomic sequences instead of artificial consensus repeats (Frebourg et al, 1992). The two p53-binding sites were constructed in tandem, proximal to a minimal c-*fos* promoter (TATA box) and a *lac*Z reporter gene. pCAT-control plasmid (Promega) was used to assay transfection efficiency.

### Transient transfection

Transfections were performed using lipofectamine (Gibco BRL). Cells were seeded into 12-well plates 24 h before transfection in order to obtain 70%. confluence. Transfection was performed according to the manufacturer's recommendations. An aliquot (5 µl) of lipofectamine was used per transfection with 0.375 µg of pRGC-Δ*fos-lac*Z DNA, 0.375 µg of p53 expression vector DNA and 0.25 µg of control plasmid DNA.

### CAT ELISA and β-galactosidase assay

After transfection with the appropriate vectors, cells were scraped and washed in PBS. CAT activity was quantified using a CAT ELISA kit (Boehringer Mannheim). Briefly, cell extracts were applied on microtitre plates coated with anti-CAT antibody. A digoxigenin-conjugated anti-CAT antibody was added as tracer. Peroxidase-conjugated anti-digoxigenin antibody and ABST substrate were used to quantify the amount of CAT protein. For the β-galactosidase assay cell extracts were incubated at 37°C in the presence of a reaction mix containing 402 µl of 0.1 M sodium phosphate pH 7.5; 132 µl of ONPG (4 mg ml$^{-1}$ in sodium phosphate 0.1 M pH 7.5) and 6 µl of 0.1 M magnesium chloride, 4.5 M β-mercaptoethanol (Maniatis et al, 1987). The reaction was stopped by adding 1 ml of 1 M sodium carbonate solution. Optical densities were read at 420 nm.

### Immunostaining

Cells were grown on coverslips and transfected with the appropriate plasmids. PAb240, PAb1801 and PAb421 monoclonal antibodies were used as a cocktail to detect p53. Cells were washed, fixed in paraformaldehyde (3% in PBS) for 15 min, preincubated with bovine serum albumin (BSA) (3% in PBS) for 30 min and further incubated for 1 h in the presence of primary antibody at room temperature. Secondary FITC-conjugated antibody (rabbit anti-mouse immunoglobulin; Dako), was added for 45 min and p53 visualized by indirect immunofluorescence.

### Detection of apoptosis

Cells stained for p53 were washed in PBS and stained with propidium iodine (Sigma 50 µg ml$^{-1}$ in PBS) for 5 min, washed twice in water, and observed under fluorescent light. Apoptosis was detected as morphological alterations of nuclear staining, counting a minimum of 150 cells.

© Cancer Research Campaign 1998

**Figure 1** Transactivation activity of wild-type p53 in different cell lines. Different cell lines were co-transfected with wt p53 expression vectors (0.375 μg), RGC–*lacZ* reporter plasmid (0.375 μg) and p-CAT–control plasmid (0.250 μg). Galactosidase activity was measured and normalized to CAT activity 60 h post-transfection. Activities are compared with wt p53 at 37°C (taken as 100%) and represent the average of four independent transfections. □, 37°C; ■, 32°C

### RESULTS

### Effects of temperature on wild-type p53

Comparison of the transcriptional activity of wt p53 in cells cultured at 37°C and at 32°C revealed a striking temperature-dependent effect. The Hep3B, HepG2 and MCF-7 cell lines all exhibited enhanced transactivation of the reporter construct when incubated at 32°C (Figure 1). This effect did not appear to reflect an effect of temperature on cell growth as the transactivation assays were performed at 60 h post-transfection and at this time cell growth rates were equivalent at 37°C and at 32°C (see Figure 2A for examples). Indeed, cell growth curves were essentially identical at both temperatures for Saos-2, HepG2 (Figure 2A) and MCF-7 cells (results not shown); for Hep3B the establishment of growth after subculture was slower at 32°C but, after 24 h, the rate of proliferation recovered and paralleled that of cells grown at 37°C (Figure 2A). The effects of temperature on cell cycle were also checked (Figure 2B: results shown are for Hep3B, other cell lines gave essentially similar results). Again, there was no obvious cell cycle effect that might account for the enhanced transcriptional activity of wt p53 at 32°C relative to 37°C.

One possible explanation for the observed effect of temperature on the transcriptional activity of p53 lies in the effect of temperature on the dissociation of p53–DNA complexes. It has been noted that the dissociation of wt p53 from a sequence-specific DNA target was markedly increased when the complexes were incubated in vitro at 30°C as opposed to 20°C (Hall and Milner, 1995; AJ Hall and J Milner, unpublished observations). If p53–DNA complexes are similarly stabilized at lower temperatures in cells, this might contribute to the enhanced transactivation effect observed at 32°C as opposed to 37°C (Figure 1). However, other factors must also be involved as the Saos-2 cells showed equivalent levels of transactivation by p53 at both 37°C and at 32°C (Figure 1). This suggests that cellular factors that are intrinsic to individual cell lines can also influence the efficacy of transactivation by wt p53 in a transient transfection assay. Such factors may be linked with cell type and/or the number of passages in culture as the Saos-2 cells used in this study had been passaged between 35–45 times, whereas the Hep3B line had been passaged 90–110 times (see Table 1). Indeed progressive loss of genetic integrity is predicted for p53-null cell lines such as Saos-2 and Hep3B.

The observed difference in the effect of temperature on transactivation by wt p53 indicates a fundamental difference between the Hep3B and Saos-2 cell lines. As both cell lines are frequently used to study the functions of p53, this observation is an important consideration when assessing and comparing results from different research groups.

### Effects of temperature on p53 mutants

We next tested the ability of selected p53 mutants to transactivate the RGC reporter gene at 30°C and at 37°C. All four cell lines (listed in Table 1) were used. The characteristics of the p53 mutant proteins after expression in vitro are listed in Table 2. In all cases the inflexible mutant, M237I, failed to show any transactivation activity (Figure 3). The two flexible mutants differed markedly in their transactivation potential after transfection into cells (Figure 3), even though they both exhibit equivalent sequence-specific DNA-binding properties in vitro (Rolley et al, 1995). Thus, M237R was similar to wt p53 in all four cell lines at 37°C. This similarity was also observed at 32°C in each cell line with the exception of HepG2, which did not exhibit a temperature-dependent effect with M237R (Figure 3).

In contrast, the second flexible mutant, V272M showed little or no transactivation at 37°C in any of the cell lines tested. In cells incubated at 32°C, V272M showed some transactivation of the RGC reporter in the two p53 null cell lines and in HepG2 cells. In vitro studies have shown that V272M exhibits equivalent sequence-specific DNA binding to the wt p53 protein (Rolley et al, 1995). Our present observation that V272M is deficient for transactivation in vivo suggests that valine at residue 272 plays a role in other molecular interactions that are important for p53 function.

### Ability of mutant p53 proteins to induce apoptosis

It has recently been reported that some mutants of p53 retain the ability to induce apoptosis, even though they have lost DNA-binding capacity and are unable to transactivate p53 target genes (Haupt and Oren, 1996). We therefore next assessed apoptosis after transient transfection with wt p53 and with p53 mutants. Hep3B cells were selected for this study as they undergo apoptosis in preference to $G_1$ arrest in response to overexpression of wt p53 (see Table 1).

As expected, wt p53 induced apoptosis in the Hep3B cells, whereas the inflexible M237I mutant failed to induce any response (Figure 4A). Interestingly, with wt p53 there was no enhanced effect at 32°C compared with 37°C. This is in contrast to the striking enhancement of transactivation activity of wt p53 at 32°C in Hep3B cells (Figure 1). The flexible mutant M237R also induced apoptosis, albeit to a lesser extent than the wt protein (Figure 4A). However, the flexible V272M mutant was inefficient at inducing apoptosis, with only 10% of those cells expressing high levels of p53 protein showing apoptotic nuclei (see Figure 4B for morphological characteristics).

### DISCUSSION

Use of transient transfection for the study of p53 protein function has proved informative for those investigations that do not require

© Cancer Research Campaign 1998 

**Figure 2** (**A**) Growth curves of HEP3B, SAOS-2 and HEPG2 cells at 37°C and 32°C. Cells were seeded into 12-well tissue culture plates at 37°C, incubated overnight, then at either 37°C or 32°C (time zero). Cell viability ($10^3$ cells per well) was estimated by trypan blue exclusion every 24 h. Open symbols represent cells at 37°C and closed symbols cells at 32°C. (**B**) Cell cycle analysis of HEP3B cells grown at 37°C and 32°C. Cells were seeded into 6-well tissue culture plates at 37°C overnight then at either 37°C or 32°C. Cell cycle analyses were performed at equivalent confluences using flowcytometry. $G_1$, S and $G_2$/M fraction were 65%, 11%, 24% and 60%, 13%, 27% at 37°C and 32°C respectively ongoing proliferation of the transfected cells (see for example the recent papers by Haupt et al, 1997, and Kubbutat et al, 1997). Established cells lines that are null for p53 are usually selected for such studies, including the human Saos-2 osteosarcoma line and Hep3B hepatocellular carcinoma cell line. Our results now demonstrate a significant difference between the functional capacity of wt p53 after transfection into Saos-2 compared with Hep3B cells. This difference is manifest by the enhanced transactivation of the RGC reporter plasmid at 32°C in Hep3B cells. The results shown are for cells assayed 60 h after transfection and represent the means of four experiments (Figure 1). Similar results were obtained 48 h after transfection (again taking the mean of four experiments, data not shown). The effects of temperature on p53 transactivation activity could not be explained by altered cell growth rate or by altered cell cycle at 32°C, and may reflect stabilization of p53–DNA complexes in conjunction with biochemical properties specific to the individual cell lines (see Results). Other factors known to impinge upon p53 in a temperature-dependent manner include heat shock protein 70 (hsp70; Hainaut and Milner, 1992 and references therein) redox and metal chelation (Hainaut and Milner 1993*a*,*b*; Verhaegh et al, 1997). Any or all these factors may contribute towards the enhanced properties of p53 in Hep3B cells cultured at 32°C. The HepG2 and MCF-7 cell lines also showed enhanced transactivation of the RGC reporter plasmid at 32°C after transfection with wt p53 (Figure 1). In these two cell lines transactivation by the endogenous wt p53 was either not detectable (HepG2) or did not appear to be affected by temperature (MCF-7; see Figure 3, controls).

In human cancer the methionine residue at position 237 of p53 can be substituted by missense point mutation to either arginine (M237R) or isoleucine (M237I). These alternative substitutions at residue 237 have strikingly different consequences at the level of

© Cancer Research Campaign 1998

**Figure 3** Transactivation activity of flexible mutants in different cell lines. Different cell lines were co-transfected with wt or mutant p53 expression vectors (0.375 μg), RGC–*lacZ* reporter plasmid (0.375 μg) and p-CAT–control plasmid (0.250 μg). Four independent transfections were performed. Galactosidase activity was measured and normalized to CAT activity 60 h post-transfection. Activities are compared with wt p53 transactivation as for Figure 1. Activity at 37°C and 32°C are represented by open bars and solid closed respectively. Control, reporter plus CMV vector protein structure. Thus, substitution to isoleucine at residue 237 completely abrogates the ability of the mutant protein to adopt the wt conformation and results in complete loss of ability either to transactivate a target gene or to induce apoptosis (Figures 3 and 4). In contrast, M237R retains the ability to adopt the wt p53 conformation and can bind a sequence-specific DNA target when translated in vitro at 30°C, whereas at 37°C it adopts the mutant conformation and fails to show sequence-specific DNA binding (Rolley et al, 1995). Our present results show that this structurally temperature-sensitive mutant is also temperature sensitive for function when transfected into Hep3B cells. Indeed, it behaves very much like the wt p53 in transient transfection studies, with wt properties at both 32°C and at 37°C (Figure 3). The ability of M237R to transactivate a target gene at 37°C indicates that cells are able to drive the conformational folding of this protein into the wt form required for sequence-specific DNA recognition and binding.

Methionine 237 is located on loop 3 of the molecular structure of the p53 core domain (Cho et al, 1994) and is close to the zinc atom that helps stabilize the tertiary conformation of wt p53. Substitution to arginine may allow similar spatial organization to the wt protein under permissive conditions. This could account for the wt-like functions of the flexible M237R mutant. Substitution to isoleucine at residue 237 is likely to disrupt this crucially important domain because of the bulky nature of isoleucine and lack of steric flexibility. This would account for the inability of M237I to adopt the wt conformation and its complete lack of functional activity.

Substitution of valine 272 with methionine (V272M) results in a p53 mutant protein that is largely indistinguishable from M237R when expressed in vitro. Both V272M and M237R are temperature sensitive for conformation and bind a sequence-specific DNA target when translated at 30°C (Rolley et al, 1995). However, we now show that the ability of V272M to transactivate a p53-specific target gene or to induce apoptosis of transfected cells is greatly impaired, whereas M237R appears similar to wt p53.

Loss of function associated with the V272M mutant is more difficult to rationalize from the molecular structure of p53. Valine

© Cancer Research Campaign 1998

**Figure 4** (**A**) Induction of apoptosis in HEP3B cells expressing wt or mutant p53. □, 37°C; ▨ 32°C. (**B**) Morphological assessment of apoptosis. Hep3B cells were transfected with wt or mutant p53 expression vectors (1 μg DNA). p53 protein expression was detected by indirect immunofluorescence and DNA staining with propidium iodine 72 h after transfection using confocal microscopy. Apoptosis was scored in p53-positive cells presenting morphological features of chromatin condensation, as shown for p53-M237R (a, arrow) compared with non-apoptotic cells expressing p53-M237I (b, arrow). Scale bars = 10 μm

272 lies on β-strand 10 within the p53 core domain (Cho et al, 1994) and is at the edge of two hydrophobic clusters. Substitution of methionine for valine at this site is unlikely to have any severe effect on conformational folding, and this is borne out by the ability of the V272M mutant to adopt the wt phenotype with sequence-specific DNA binding when expressed at 30°C in vitro (Rolley et al, 1995). Nonetheless, V272M is clearly grossly impaired for normal functioning in the cell (Results), implying that V272 may be important for intramolecular interactions involved in p53 transactivation and induction of apoptosis.

Our results indicate that the functional properties of wt p53 can vary with temperature and with cell line when the protein is over-expressed after transient transfection. In particular, Saos-2 and Hep3B differ markedly and it is possible that these two p53-null cell lines may have diverged because of genomic instability in the absence of p53. It is equally possible that established p53-null lines derived from Saos-2, for example, may also differ between different laboratories depending on passage number. Indeed, in the case of Hep3B we have evidence that the functioning of certain p53 mutants vary according to cell passage number (F Ponchel and J Milner, unpublished observations). It follows that use of p53-null cells to study the functional properties of p53 should ideally be restricted to primary cells and cell lines with low passage number.

In summary, we believe that our results have important implications for experimental model systems currently used to study the functions of wild-type and mutant p53 proteins. In particular, we emphasize the observed variation between different p53-null cell lines, that may, in part, reflect the inherent genetic instability of such cell lines.

It is hoped that information gained from the study of p53 in cell culture will, in the longer term, prove useful for the development of novel therapies for the treatment of cancer. The first message from our present study is that it is not sufficient to simply classify p53 in tumour cells as either wild type or mutant. Not all mutants

 © Cancer Research Campaign 1998

cause complete loss of p53 function and there is a realistic possibility of rescuing wt function for many clinically relevant p53 mutants. In our own studies we have screened for mutants able to function at lowered temperatures. However, we are unable to discuss our observations in relation to current clinical therapies as there is little published information on the use of hypothermia in cancer therapy. Hyperthermia, on the other hand, has been used in conjunction with radiotherapy for the treatment of certain tumour types. Based on observations on the behaviour of both wt and mutant p53 proteins at different temperatures (Hainaut et al, 1995*b*, Rolley et al, 1995; Friedlander et al, 1996*b*; Hansen et al, 1996; this paper) we would predict minimal involvement of p53 under hyperthermic conditions. This would be consistent with the observed lack of correlation between p53 genetic status and effects of preoperative radiochemohyperthermia therapy for rectal cancer (Ichikawa et al, 1996).

There is evidence that cellular factors can influence restoration of wt functions to certain p53 mutants (see above) and future work aims to identify these factors. Such information should lead to informed screening for potential therapeutic agents that may similarly enhance the functional capacity of mutant p53 proteins. It should also be remembered that the ability of p53 to transactivate target gene expression is not essential for its ability to induce apoptosis (Haupt et al, 1995). Thus, for some mutants, it may be possible to activate p53-dependent apoptotic pathways (even though their transactivation potential has been irrevocably lost) and so induce selective killing of the affected tumour cells.

### ACKNOWLEDGEMENTS

We thank Dr. Carlos Rubbi for help in preparation of Figure 4B and Dr. Andrei Okorokov (YCRC P53 Research Group) for help in the analysis of the molecular structure of p53 and for critical reading of the manuscript. This work was funded by the Yorkshire Cancer Research Campaign and by HMG EC funding CT93-0180 (to JM).

### REFERENCES

Aden DP, Fogel A, Plotkin S, Damjanov I and Knowles BB (1979) Controlled synthesis of HBsAg in a differentiated human liver carcinoma-derived cell line. *Nature* **282**: 615–616

Canman C and Kastan M (1995) Induction of apoptosis by tumor-suppressor genes and oncogenes. *Sem Cancer Biol* **6**: 17–25

Cho Y, Gorina S, Jeffrey PD and Pavletich NP (1994) Crystal structure of a p53 tumor suppressor-DNA complex: understanding tumorigenic mutations. *Science* **265**: 346–355

Fogh J and Trempe G (1975) *Human Tumor Cells In Vitro.* pp. 115–159. Plenum Press: New York

Forrester K, Lupold S, Ott V, Chay C, Band V, Wang X and Harris C (1995) Effects of p53 mutant on wild type p53-mediated transactivation are cell type dependent. *Oncogene* **10**: 2103–2111

Frebourg T, Barbier N, Kassel J, Ng YS, Romero P and Friend SH (1992) A functional screen for germ line p53 mutations based on transcriptional activation. *Cancer Res* **52**: 6976–6978

Freidman S, Shaulian E, Littlewood T, Resnitzky D and Oren M (1997) Resistance to p53-mediated growth arrest and apoptosis in Hep3B hepatoma cells. *Oncogene* **15**: 63–70

Friedlander P, Prives C and Oren M (1996*a*) A mutant p53 that discriminates between p53-responsive genes cannot induce apoptosis. *Mol Cell Biol* **16**: 4961–4971

Friedlander P, Soussi T and Prives C (1996*b*) Regulation of mutant p53 temperature-sensitive DNA-binding. *J Biol Chem* **271**: 25468–25478

Guillot C, Courtois S, Voeltzel T, Garcia E, Ozturk M and Puisieux A (1996) Alteration of p53 damage response by tamoxifen treatment. *Clin Cancer Res* **2**: 1439–1444

Hainaut P and Milner J (1992) Interaction of heat-shock protein 70 with p53 translated in vitro: evidence for interaction with dimeric p53 and for a role in the regulation of p53 conformation. *EMBO J* **11**: 3513–3520

Hainaut P and Milner J (1993*a*) Redox modulation of p53 conformation and sequence-specific DNA binding in vitro. *Cancer Res* **59**: 4469–4473

Hainaut P and Milner J (1993*b*) A structural role for metal ions in the 'wild-type' conformation of the tumor suppressor protein p53. *Cancer Res* **53**: 1739–1742

Hainaut P (1995*a*) The tumor suppressor protein p53: a receptor to genotoxic stress that controls cell growth and survival. *Curr Opin Oncol* **7**: 76–82

Hainaut P, Butcher S and Milner J (1995*b*) Temperature sensitivity for conformation is an intrinsic property of wild type p53. *Br J Cancer* **71**: 227–231

Hall AJ and Milner J (1995) Structural and kinetic-analysis of p53–DNA complexes and comparison of human and murine p53. *Oncogene* **10**: 561–567

Hansen S, Hupp T and Lane D (1996) Allosteric regulation of the thermostability and DNA-binding activity of human p53 by specific interacting proteins. *J Biol Chem* **271**: 3917–3924

Haupt Y and Oren M (1996) Cell-type-specific inhibition of P53-mediated apoptosis by mdm2. *EMBO J* **15**: 1596–1606

Haupt Y, Rowan S, Shaulian E, Vousden K and Oren M (1995) Induction of apoptosis in HeLa cells by transactivation-deficient p53. *Genes Dev* **9:** 2170–2183

Haupt Y, Maya R, Kazaz A and Oren M (1997) Mdm-2 promotes the rapid degradation of p53. *Nature* **387**: 296–298

Ichikawa D, Yamaguchi T, Shirasu M, Kitamura K, Inazawa J, Abe T and Takahasi T (1996). p53 gene mutation is not directly related to tumoricidal effects of preoperative radiochemohyperthermia therapy for rectal cancers. *J Surg Oncol* **63**: 89–90

Jiang M, Lin J and Yen J (1996) Differential regulation of p53, c-Myc, Bcl-2 and Bax protein expression during apoptosis induced by widely divergent stimuli in human hepatoblastoma cells. *Oncogene* **13:** 609–616

Ko LJ and Prives C (1996) P53 – puzzle and paradigm. *Genes Dev* **10**: 1054–1072.

Kubbutat M, Jones S and Vousden K (1997) Regulation of p53 stability by mdm-2. *Nature* **387**: 299–302

Maniatis T, Goodbourn S and Fischer JA (1987) Regulation of inducible and tissue-specific gene expression. *Science* **236**: 1237–1244

Medcalf EA, Takahashi T, Chiba I, Minna J and Milner J (1992) Temperature-sensitive mutants of p53 associated with human carcinoma of the lung. *Oncogene* **7**: 71–76

Michalovitz D, Halevy O and Oren M (1990) Conditional inhibition of transformation and of cell proliferation by a temperature-sensitive mutant of p53. *Cell* **62**: 671–680

Milner J (1995) DNA damage, p53 and anticancer therapies. *Nature Med* **1**: 879–880

Milner J and Medcalf EA (1990). Temperature-dependent switching between 'wild-type' and 'mutant' forms of p53-Val135. *J Mol Biol* **216**: 481–484

Muller M, Strand S, Hug H, Heinemann E, Walczak H, Hoffmann W, Stremmel W, Kramer P and Galle P (1997) Drug induced apoptosis in hepatoma cells is mediated by the CD95 (APO-1/Fas) receptor/ligand system and involves activation of wild type p53. *J Clin Invest* **99**: 403–413

Ogretmen B and Safa A (1997) Expression of the mutated p53 tumor suppressor protein and its molecular and biochemical characterisation in multidrug resistant MCF-7/Adr human breast cancer cells. *Oncogene* **14**: 499–506

Ponchel F, Puisieux A, Tabone E, Michot JP, Froschl G, Morel AP, Frebourg T, Fontaniere B, Oberhammer F and Ozturk M (1994) Hepatocarcinoma-specific mutant p53-249ser induces mitotic activity but has no effect on transforming growth factor beta 1-mediated apoptosis. *Cancer Res* **54**: 2064–2068

Rolley N, Butcher S and Milner J (1995) Specific DNA binding by different classes of human p53 mutant. *Oncogene* **11**: 763–770

Soule H (1973) *J Natl Cancer Inst* **51**: 1409–1416

Verhgaegh W, Richard M and Hainaut P (1997) Regulation of p53 by metal ion and antioxydant:dithiocarbanate down regulate p53-DNA binding activity by increasing the intracellular level of copper. *Mol Cell Biol* **17**: 5699–5706

Yamato K, Hirano Y and Tsuchida N (1995) A human temperature-sensitive p53 mutant P53 (Val-138) – modulation of the cell-cycle, viability and expression of p53-responsive genes. *Oncogene* **11**: 1–6

© Cancer Research Campaign 1998